# Artificial intelligence for risk assessment and outcome prediction in malignant haematology

**DOI:** 10.1111/bjh.70231

**Published:** 2025-11-13

**Authors:** Jan‐Niklas Eckardt, Wencke Walter, Jan Moritz Middeke, Torsten Haferlach

**Affiliations:** ^1^ Department of Internal Medicine I, University Hospital Carl Gustav Carus TUD Dresden University of Technology Dresden Germany; ^2^ Else Kröner Fresenius Center for Digital Health TUD Dresden University of Technology Dresden Germany; ^3^ MLL Munich Leukemia Laboratory Munich Germany

**Keywords:** artificial intelligence, deep learning, machine learning, outcome prediction, risk stratification

## Abstract

Risk stratification is the backbone of clinical decision‐making for patients with haematological malignancies. Models currently used in routine clinical practice often rely on static rules, conventional statistics and expert consensus. While pragmatic and accessible, these models fail to capture the complex biological interactions and patient heterogeneity, especially as data dimensionality grows given the availability of more comprehensive molecular analysis. Artificial intelligence (AI), particularly machine learning (ML), offers the potential to process such high‐dimensional data, uncover latent patterns and provide dynamic, individualized risk predictions. In this review, we introduce haematologists to core concepts and limitations of AI‐based risk stratification. We examine studies in myeloid and lymphoid neoplasms developing AI models that challenge current standard models and identify novel disease subtypes and risk markers. While these models offer unique potential for data‐driven personalized decision support, several challenges remain to routine applicability such as training cohort composition, validation and bias mitigation highlighting an urgent need for interdisciplinary collaboration and dialogue with regulatory agencies to ensure robust and safe model deployment. As data dimensionality, digital infrastructure and regulatory frameworks evolve, AI‐guided risk stratification is poised to complement and potentially reshape clinical practice in malignant haematology requiring haematologists to be aware of potential capabilities and pitfalls.

## Graphical abstract

Machine learning models allow for dynamic and scalable risk stratification and outcome prediction. Different modalities of data such as electronic health records, patient genetics or laboratory results can be used as input. ML models autonomously select features weighing their prognostic value. Methods of model explainability in feature selection allow for expert validation and enable identification of novel biomarkers.

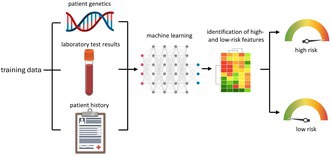

## INTRODUCTION

Risk stratification in malignant haematology aims to guide haematologists in making treatment decisions weighing individual disease biology and patient‐specific factors. Depending on the disease, there is a multitude of models that are currently considered the gold standard in clinical routine such as the European LeukemiaNet (ELN)^1^ model in acute myeloid leukaemia (AML), the Revised International Prognostic Scoring System (IPSS‐R)^2^ in myelodysplastic neoplasms (MDS) or the International Prognostic Index (IPI)^3^ in diffuse large B‐cell lymphoma (DLBCL). These systems have proven their worth over decades. Their basis often is routine clinical or genetic data, guided by conventional statistical modelling and expert consensus. In clinical routine, they provide the means for pragmatic decision support as they essentially function as checklists or simple decision trees that can be memorized without assigning complex weights to their individual variables. Yet, haematological malignancies are inherently heterogeneous and often characterized by multiple concurrent aberrations. The current static approaches are likely unable to fully appreciate the complex interplay in disease biology. Given the increasing access to next‐generation sequencing or whole‐exome/genome and transcriptomic profiling, the dimensionality of patient‐specific data is going to rapidly outpace the interpretive capacity of conventional rule‐based systems. This introduces an urgent need for more flexible, data‐driven models capable of handling high‐dimensional datasets to integrate disease biology and patient‐specific factors.

Machine learning (ML) may address this challenge better than static rule‐based approaches. While initial applications of ML in haematology emerged over two decades ago,^4^, ^5^, ^6^, ^7^ early efforts were constrained by limited data integration and computational resources. In recent years, advances in algorithms, computational power, and access to large, multimodal datasets have brought AI‐guided clinical decision support systems within reach.^8^, ^9^ These systems can be trained to identify complex patterns and interactions in heterogeneous datasets and offer outcome predictions and risk stratification that may surpass traditional models both in accuracy and scale.

Importantly, these systems are not intended to replace clinical expertise but to augment it by supporting clinicians in evaluating vast and multifaceted patient data and informing personalized evidence‐based care. However, their implementation also introduces new challenges including model transparency, bias mitigation, validation, regulation and clinical integration. An understanding of the capabilities and limitations of AI‐driven decision support tools is essential for both clinicians and researchers in haematology. Ensuring that these technologies enhance rather than complicate patient care will depend on rigorous assessment, safe clinical integration and continued interdisciplinary dialogue between haematology and computational sciences.

In this review, we aim to equip haematologists with an understanding of both capabilities and limitations of ML models for risk prediction, highlight studies using ML‐based risk prediction across malignant haematology and discuss emerging trends and potential pitfalls for the clinical integration of these tools in the upcoming years.

## A BRIEF INTRODUCTION TO MACHINE LEARNING FOR HAEMATOLOGISTS

The terms ‘artificial intelligence’ and ‘machine learning’ have become ubiquitous in healthcare. Despite widespread exposure, understanding of AI and ML varies significantly. A survey^10^ showed that 21.9% of self‐reported ML‐literate respondents misidentified its definition, likely owing to the overlapping use of AI terminology in medicine.

AI broadly refers to digital problem‐solving techniques that would otherwise require human intelligence.^11^ ML, a subset of AI, is focused on developing algorithms that learn from data to make predictions or uncover patterns,^11^ setting it apart from traditional software, which operates based on explicit instructions. These models scale with their training data, which make them particularly suitable for handling the high‐dimensional datasets encountered in haematology, including molecular profiles, clinical variables and laboratory parameters. Within ML, deep learning (DL) utilizes neural networks that mimic information processing in the neocortex via interconnected artificial neurons (also termed *perceptrons*).^12^ Much like neural plasticity, the weights of the connections between these neurons are calibrated in the training stage, enabling neural networks to process large amounts of data such as images or text.^12^ Convolutional neural networks (CNNs) were initially considered state of the art in image processing^13^; however, the introduction of the transformer architecture (incorporating multi‐head attention)^14^ revolutionized image and text processing, enabling the development of large language models and foundation models with increasing multimodal capabilities such as text, image or speech processing.

At the core of ML is model inference through methods like supervised learning, unsupervised learning and reinforcement learning.^11^ Supervised learning, which pairs input data (e.g. genetic profile, differential count, pharmacogenomics data) with corresponding output labels (e.g. disease subtype, treatment outcome), is particularly suited to risk prediction tasks in haematology. It allows models to forecast specific outcomes based on patient data. Unsupervised learning, which does not rely on labelled outcomes, is invaluable for discovering new insights into disease mechanisms or patient stratification. In unsupervised learning, data are sorted by similarity or dissimilarity, allowing for cluster formation of patients with biologically related subgroups of a given entity. Reinforcement learning (RL) is an iterative method that links decision‐making to changes in outcomes or states of a simulated environment, thereby aiming at developing an optimal strategy to achieve a predefined goal. Since RL explicitly requires ML models to alter their environment, that is, make actual treatment decisions in the medical context, it is currently not used for direct risk prediction but can optimize treatment strategies or clinical pathways based on patient outcomes, for instance, in simulated environments.^15^ The nature of rare data in medicine often requires pooling and harmonizing datasets from different institutions. If sharing data are not feasible, techniques like federated learning^16^ where the model is shared between collaborators or swarm learning^17^ where only model weights (learnable parameters) are shared offer alternatives that enable collaborative model development without sharing data.

Evaluating the performance of risk prediction models is crucial, particularly in haematology, where outcomes can significantly impact patient well‐being and survival. Sensitivity measures the proportion of actual high‐risk patients correctly identified, while specificity measures the proportion of truly low‐risk patients (or non‐events) correctly classified. High specificity reduces the number of false alarms, which can be important to avoid unnecessary treatment or interventions. The area under the receiver operating characteristic (AUC‐ROC) is particularly valuable for risk prediction models because it provides a comprehensive measure of the model's ability to distinguish between different outcomes (e.g. high risk vs. low risk) across all possible thresholds. A higher AUC indicates better model performance, with a score of 1 representing a perfect model and a score of 0.5 indicating no discriminative ability. Datasets in haematology are often imbalanced, with certain genetic or phenotypic characteristics being more common than others or certain events may occur rarely. In these cases, AUC‐ROC may be misleading and the precision–recall curve and its area under the curve (AUC‐PR) become important. These metrics focus on the model's ability to retrieve relevant instances (recall) ensuring that among patients predicted as high risk, a high proportion truly is high risk (precision). F1 score balances precision and recall as a harmonic mean. It is useful especially in settings where both missing high‐risk patients and overpredicting risk carry consequences. The concordance‐index (c‐index) evaluates the performance of survival models and any predictive model where the outcome is time until an event occurs. It provides an effective measure of a model's discriminative capacity in scenarios where the timing of events is critical. Beyond the discrimination of outcomes, clinical risk prediction models must also demonstrate good calibration, that is, the agreement between predicted and observed event probabilities.^18^ A well‐calibrated model ensures that, for example, patients predicted to have a 20% probability of relapse experience relapse in approximately 20% of cases. Poor calibration can result in over‐ or underestimation of absolute risk and may therefore misguide clinical decisions even when discrimination performance is high.^19^ Calibration can be evaluated, for example, using calibration plots or the Brier score^20^ and can be improved via, for example, isotonic or Platt scaling.^21^ Ideally, both discrimination performance as well as calibration should be reported for models intended to guide clinical decisions. In practice, no single metric suffices; however, combining several complementary metrics usually provides the most reliable picture of a model's performance.

A common pitfall of ML prediction models is overfitting, which describes the notion that a model does adequately learn patterns during training but rather memorizes the training data. Upon evaluating new previously unseen data, model performance subsequently plummets, and the model is not transferable or generalizable to other data within the scope it was trained for. Therefore, overfitting can be assumed if a model performs well on training data but performance drops in internal or external validation sets. Large, diverse datasets, independent validation sets, techniques such as cross‐validation, dropout, regularization, dimensionality reduction (i.e. reducing the number of variables relative to sample size) or alterations to model architecture may help mitigate or detect overfitting.^22^ Table 1 summarizes key AI terminology.

**TABLE 1:** Key terminology in artificial intelligence.

Key term	Description
Artificial intelligence (AI)	Broad field of computer science aimed at creating systems capable of performing tasks that normally require human intelligence, such as pattern recognition, forecasting or decision‐making.
Machine learning (ML)	Subfield of AI where algorithms learn from data to make predictions or decisions without being explicitly programmed.
Deep learning (DL)	A form of ML using multilayer neural networks that can learn complex patterns from large datasets such as genomics or imaging data.
Supervised learning	Category of ML where a model is trained on labelled data (inputs with known outcomes) to predict outcomes in new cases.
Unsupervised learning	Category of ML where a model identifies structures or clusters in unlabelled data, revealing subgroups or new biological patterns.
Reinforcement learning (RL)	Category of ML where a model learns through trial‐and‐error interaction with a simulated environment to optimize decisions; may be used to test treatment strategies.
Neural network (NN)	Computational architecture inspired by the structure of the neocortex, composed of layers of interconnected artificial neurons (alternatively *perceptrons*) with learnable weights (connections between the neurons).
Convolutional neural network (CNN)	Type of neural network excelling in image analysis (e.g. blood or bone marrow microscopy).
Transformer	Neural network architecture is designed to process sequential data (text, genomic sequences) using attention mechanisms rather than recurrence, being the foundation of modern large language models.
Large language model (LLM)	Transformer‐based model trained on massive text datasets to understand and generate human language. LLMs can be adapted to biomedical texts for literature synthesis or report generation.
Foundation model	Very large model trained on diverse, multimodal datasets (text, images, structured data) that can be fine‐tuned for a wide range of downstream tasks, including clinical prediction, image analysis or report summarization.
Overfitting	Instead of learning patterns from the data, the model essentially memorizes the training data, leading to high performance on training sets, but inadequate performance on (external) test data, indicating low generalizability.
Regularization	Mathematical strategies (e.g. L1/L2 penalties, dropout) to prevent overfitting by constraining model complexity.
Calibration	Agreement between predicted probabilities and actual outcomes which is essential for trustworthy clinical risk prediction.
Federated learning	Decentralized technique where models are trained across institutions without sharing raw patient data, preserving privacy.
Swarm learning	Decentralized technique where model weights are shared across institutions without sharing raw patient data or the model itself, preserving privacy.
Explainability (XAI)	Techniques that make model reasoning interpretable (e.g. SHAP values, attention maps), enabling clinicians to understand which features drive predictions.

## RISK PREDICTION WITH MACHINE LEARNING IN MYELOID NEOPLASMS

### Myelodysplastic neoplasms

Early diagnosis of myelodysplastic neoplasms (MDS) enables physicians to decide about any optional treatment sooner, potentially delaying the progression of MDS and enhancing the patient's quality of life. Radhachandran et al.^23^ developed an ML algorithm using 2 years of patient data from electronic health records (EHR) to predict MDS 1 year before diagnosis, achieving an AUROC of 0.87. The model identified the patient's age, haematocrit, red blood cell count and platelet count as the most significant predictors. Clichet et al.^24^ also focused on predicting the risk of MDS but utilized multiparametric flow cytometry data instead. Their elasticnet algorithm, trained on data from 191 patients and externally validated on 89 patients, demonstrated an AUROC of 0.935, also distinguishing between high‐ and low‐risk MDS. Addressing MDS risk classification, Nazha et al.^25^ introduced an unsupervised personalized prediction model that incorporates genetic alterations, laboratory results, WHO 2016 subtypes and demographic data. This model estimates the survival and the probability of leukaemia transformation. More recently, Mosquera‐Orgueira et al.^26^ developed the Artificial Intelligence Prognostic Scoring System for MDS (AIPSS‐MDS), which relies on traditional clinical, haematological and cytogenetic variables. ML models have also shown promise in predicting treatment responses based on various genetic and clinical factors. Nazha et al.^27^ applied Apriori market basket analysis to identify genomic biomarkers predictive of resistance to hypomethylating agents (HMA) in MDS patients. Expanding on this, Radakovich et al.^28^ created an ML model that assesses the response to HMA by analysing serial blood counts during the first 90 days of treatment. After 3 months, this model could predict HMA response with an AUROC of 0.84 in an independent validation cohort. Similarly, Sharplin et al.^29^ developed a random forest model to forecast early treatment failure in patients receiving azacitidine. Table 2 summarizes studies investigating ML for risk prediction in MDS.

**TABLE 2:** Studies investigating ML‐based outcome prediction in myelodysplastic neoplasms (MDS).

References	Sample(s)	ML model(s)	Output	Validation
Radhachandran et al.^23^	MDS (*n* = 790 470)	XGB	MDS risk prediction 1 year prior to diagnosis, AUROC 0.87	Internal, hold‐out
Clichet et al.^24^	MDS (*n* = 191)	Elasticnet	Risk of MDS	External (*n* = 89)
Nazha et al.^25^	MDS (*n* = 1471)	RSF	OS and risk of leukaemia transformation	External (*n* = 465)
Mosquera‐Orgueira et al.^26^	MDS (*n* = 7202)	RSF	OS and LFS	External (*n* = 2957)
Nazha et al.^27^	MDS with HMA treatment (*n* = 433)	Apriori market basket analysis	Risk of HMA resistance	External (*n* = 113)
Radakovich et al.^28^	MDS (*n* = 424)	Random Forest, gradient‐boosted decision trees	HMA response	External (*n* = 90)
Sharplin et al.^29^	MDS, AML, CMML treated with azacitidine (*n* = 273)	RSF	Risk of treatment failure, survival outcome	Internal, cross‐validation

Abbreviations: AML, acute myeloid leukaemia; AUROC, area under the receiver operating characteristic; CMML, chronic myelomonocytic leukaemia; HMA, hypomethylating agents; LFS, leukaemia‐free survival; OS, overall survival; RF, random forest; RSF, random survival forest; XGB, extreme gradient boosting.

### Myeloproliferative neoplasms

Myeloproliferative neoplasms (MPN) are phenotypically very heterogeneous, with an inherent risk of disease progression. Chronic myeloid leukaemia (CML) can be effectively treated with tyrosine kinase inhibitors (TKI). Nevertheless, TKI response and outcome prediction are paramount. Shanbehzadeh et al.^30^ evaluated multiple ML methods and identified SVM as the most accurate method for predicting 5‐year survival in CML patients, based on 12 clinical features. In myelofibrosis, Mosquera‐Orgueira et al.^31^ developed an Artificial Intelligence Prognostic Scoring System for Myelofibrosis (AIPSS‐MF) model, based on eight clinical variables at diagnosis, which achieved high accuracy in predicting overall survival (OS) and leukaemia‐free survival (LFS) for myelofibrosis patients. The grading of fibrosis is an important component of disease classification, prognostication and monitoring. Ryou et al.^32^ developed an ML method to objectively assess bone marrow reticulin fibrosis, creating a Continuous Index of Fibrosis (CIF) using manually labelled fibrosis areas for training. This approach, which includes a quantitative fibrosis map and megakaryocyte analysis, accurately differentiates between ET and pre‐fibrotic MF (AUC = 0.94) and identifies MPN patients at risk of disease progression. With the incorporation of effective therapies for MF, accurately predicting outcomes after allogeneic haematopoietic cell transplantation (allo‐HCT) is crucial for determining the optimal timing for this procedure. Hernández‐Boluda et al.^33^ created a random survival forest (RSF) model to identify patients at high risk for poor transplantation outcomes. Table 3 summarizes studies investigating ML for risk prediction in MPN.

**TABLE 3:** Studies investigating ML‐based outcome prediction in myeloproliferative neoplasms (MPN).

References	Sample(s)	ML model(s)	Output	Validation
Shanbehzadeh et al.^30^	CML (*n* = 837)	SVM	CML 5‐year survival, accuracy 85.7%	Internal cross‐validation
Mosquera‐Orgueira et al.^31^	MF (*n* = 1386)	RF	OS and LFS	Internal cross‐validation
Ryou et al.^32^	MPN, reactive marrow (*n* = 107)	Ranking‐CNN, RF	MPN patients at risk of progression	‐
Hernández‐Boluda et al.^33^	MF (*n* = 5183)	RSF	OS after transplant	‐

Abbreviations: CML, chronic myeloid leukaemia; CNN, convolutional neural network; LFS, leukaemia‐free survival; MF, myelofibrosis; OS, overall survival; RF, random forest; RSF, random survival forest; SVM, support vector machine.

### Acute myeloid leukaemia

Acute myeloid leukaemia (AML) is a severe haematological malignancy characterized by its heterogeneity in clinical presentation, genetic landscape and prognosis. This variability presents a significant challenge in predicting patient outcomes, with some subgroups showing potential for cure while others face poor prognoses under current treatment standards. Shaik et al.^34^ developed an ML‐based genomic‐driven prognostication model for AML patients with the *RUNX1::RUNX1T1* fusion. This model demonstrated a strong correlation with measurable residual disease (MRD) and clinical outcomes, highlighting the potential of ML to uncover prognostic markers that are clinically relevant. Furthermore, the same group^35^ introduced a supervised ML algorithm aimed at prognosticating AML with mutated *NPM1* based on genetic profiles. Despite *NPM1* mutations defining a distinct AML subgroup, these mutations do not always serve as the initiating event, underscoring the complexity of AML's genetic underpinnings. The concept of age‐related clonal haematopoiesis (ARCH) as a precursor to myeloid malignancies, including in the *NPM1* AML subgroup, adds another layer of complexity to prognostication. Patkar et al.^35^ employed a computational approach to decipher the clinical significance of numerous genetic variables. They identified that the type of *NPM1* mutation, the corrected *NPM1* variant allele fraction (VAF), the presence of *DNMT3A* R882 mutation, *FLT3* internal tandem duplication VAF and *IDH2* mutations were all clinically relevant. From these ML‐derived variables, a scoring system was developed, capable of classifying AML with mutated *NPM1* into three prognostic classes with significantly different outcomes.

Going in a similar direction, Eckardt et al.^36^ explored how age influences the prognostic significance of genetic mutations in AML. They developed ML models that effectively predicted complete remission and 2‐year overall survival, with AUC scores of 0.801 and 0.791 respectively. By employing Shapley (SHAP) values, the study quantified each variable's contribution across different age groups, revealing significant variability in the prognostic value of genetic alterations with age. This finding challenges the uniform application of risk stratification models and underscores the necessity for age‐adjusted treatment strategies in AML. Eckardt et al.^37^ also utilized nine ML models to predict complete remission and 2‐year overall survival in intensively treated AML patients, achieving high accuracies for both internal testing and external validation. The achievement of complete remission is a crucial milestone in AML therapy, as refractory disease is associated with dismal outcomes. Thus, accurately identifying patients at risk is essential for tailoring treatment concepts to individual disease biology. Using unsupervised learning, the same group^38^ identified four distinct biological clusters in AML re‐stratifying patients regarding ELN2017 risk assignment. Clusters differed significantly regarding both molecular alterations present per cluster and outcomes and were validated in an external cohort. Gal et al.^39^ applied three different ML algorithms using pretreatment gene expression data to predict complete remission in intensively treated AML patients. Pathway enrichment analysis for the most significantly deregulated genes indicated the guanosine diphosphate fucose biosynthesis pathway as the most significant, suggesting a crucial role for N‐glycosylation in AML. Wagner et al.^40^ identified a three‐gene signature (*CALCRL, CD109, LSP1*) that distinctively predicted event‐free survival (EFS) and overall survival (OS) in AML patients. This signature, validated in public AML cohorts comprising 1050 patients (adult and paediatric), was found to be uniquely predictive of survival in AML among 39 distinct malignancies, further establishing ML's potential in identifying novel prognostic markers. In a smaller study, Karami et al.^41^ utilized six different ML algorithms to predict OS in AML patients, identifying the gradient boosted tree as the most reliable method. Most recently, Jia et al.^42^ employed 11 ML models to predict survival status at 1, 2 and 3 years after AML diagnosis based on epidemiological characteristics in an overarching study that included 87 090 patients, showcasing ML's scalability and adaptability in handling large datasets for survival prediction. Table 4 summarizes studies investigating ML for risk prediction in AML.

**TABLE 4:** Studies investigating ML‐based outcome prediction in acute myeloid leukaemia (AML).

References	Sample(s)	ML model(s)	Output	Validation
Shaik et al.^34^	AML *RUNX1::RUNX1T1* (*n* = 131)	LR	Two risk classes with divergent OS & RSF	Internal, validation cohort
Patkar et al.^35^	AML NPM1 (*n* = 110)	LR	Three AML NPM1 risk groups with divergent OS	‐
Eckardt et al.^36^	AML (*n* = 3062)	RF, LR, XGB	Likelihood of CR, 2‐year OS, age‐stratified prognostic variables	Internal
Eckardt et al.^37^	AML (*n* = 1383)	SVM, RF, LR, RBF‐SVM, ANN, adaptive boosting, pSVM, gradient boosting, KNN	Likelihood of CR, 2‐year OS	External (*n* = 664)
Eckardt et al.^38^	AML (*n* = 1383)	Meta‐clustering of 11 different clustering algorithms	Risk assignment based on molecular and cytogenetics, comparison to ELN 2017	External (*n* = 664)
Gal et al.^39^	AML (*n* = 473)	KNN, RF, SVM	Likelihood of CR	Internal, cross‐validation
Wagner et al.^40^	AML (*n* = 593)	ANN	EFS, OS, 3‐gene expression signature	External, public cohorts (*n* = 1050)
Karami et al.^41^	AML (*n* = 249)	RF, DT, LR, Naive Bayes, W‐Bayes Net, GBT	OS	Internal, cross‐validation
Jia et al.^42^	AML (*n* = 87 090)	XGB, glmnet, KNN, LR, Naive Bayes, NN, RF, SVM, LDA, QDA, DT	1‐,2‐,3‐year OS	Internal, cross‐validation

Abbreviations: ANN, artificial neural net; DT, decision tree; GBT, gradient boosting tree; KNN, k nearest neighbour; LDA, linear discriminant analysis; LR, logistic regression; NN, neural network; pSVM, polynomial support vector machine; QDA, quadratic discriminant analysis; RBF‐SVM, radial basis kernel function support vector machine; RF, random forest; SVM, support vector machine; W‐Bayes Net, weighted Bayesian Network; XGB, extreme gradient boosting.

## RISK PREDICTION WITH MACHINE LEARNING IN LYMPHOID NEOPLASMS

### Diffuse large B‐cell lymphoma

In diffuse large B‐cell lymphoma (DLBCL), multiple studies investigated divergent biological subgroups beyond the conventional cell‐of‐origin classification (COO).^43^ Early studies by Shipp et al.^44^ delineated follicular lymphoma (FL) and DLBCL and predicted response to CHOP‐based regimens using a supervised approach and identified two groups with distinct 5‐year OS rates. In comprehensive genomic analyses, both Chapuy et al.^45^ and Schmitz et al.^46^ identified five and four (partially overlapping) genetic driver subgroups with impact on 5‐year OS rates, including favourable risk ABC and poor‐risk GCB subtypes, thereby highlighting the need for genetic stratification beyond COO. Zhuang et al.^47^ used unsupervised clustering to identify two subgroups with divergent survival (immune‐enriched and cell cycle‐enriched) in a cohort of 414 DLBCL patients using comprehensive gene expression data and subsequently constructed a classifier that sorted patients into their proposed subgroups using only seven divergent genes. Similarly, He et al.^48^ used consensus clustering on gene expression data and identified two clusters differing by their expression of programmed cell death‐related genes, identifying *SCARB2*, *GBP1* and *STOM* as highly relevant genes across models predicting expression patterns. Comparing a stacked model‐based outcome prediction with routine scores, Biccler et al.^49^ used data from the Danish and Swedish lymphoma registries (including clinicopathological data, treatments and outcomes) and reported superior performance of their model (validation set c‐index 0.74) outperforming both IPI (c‐index 0.66) and NCCN‐IPI (c‐index 0.68) in predicting patient survival. Mosqueira Orgueira et al.^50^ used identified two clusters delineated by their differential expression of *TNFRSF9, BIRC3, BCL2L1* and *G3BP2*. Subsequently, they developed a random survival forest‐based classifier including clinical information, COO classification, their proposed four‐gene expression cluster assignment and gene expression data, reporting a test set c‐index of 0.79 for survival prediction. Furthermore, using demographic and clinical information, Detrait et al.^51^ evaluated five ML classifiers in predicting primary refractory disease, among which naïve Bayes (NB) achieved the highest classification performance with an F1‐score of 0.82. Features with the highest importance for model decisions were IPI score, age > 80 years, high body mass index, time to treatment and PET‐CT scan performed after two cycles of treatment. As comprehensive genomic analyses are often not available in clinical routine, several studies investigated COO prediction in DLBCL with a reduced set of input variables. Zhao et al.^52^ identified eight genes (*MYBL1, LMO2, BCL6, MME, IRF4, NFKBIZ, PDE4B* and *SLA*) to predict either GCB or non‐GCB subtypes with 91.0–94.4% validation accuracy. Similarly, Viswanathan et al.^53^ used a multilayer perceptron‐based model to predict COO using gene expression data of 20 genes, achieving a test set and external validation accuracy of 94.7% and 95.5%, respectively, demonstrating close alignment between survival predictions of their classifier and conventional COO subtyping. Table 5 summarizes studies investigating ML for risk prediction in DLBCL.

**TABLE 5:** Studies investigating ML‐based outcome prediction in diffuse large B‐cell lymphoma (DLBCL).

References	Sample(s)	ML model(s)	Output	Validation
Shipp et al.^44^	DLBCL (*n* = 58) FL (*n* = 19)	Weighted voting (supervised)	Identified two patient groups with 70% vs. 12% 5‐y OS	Internal cross‐validation
Chapuy et al.^45^	DLBCL (*n* = 304)	Non‐negative matrix factorization consensus clustering	Identified 5 clusters with driver genetics and divergent outcomes	‐
Schmitz et al.^46^	DLBCL (*n* = 574)	RF, SVM, GenClass	Identified 4 with driver genetics and divergent outcomes	Internal cross‐validation
Zhuang et al.^47^	DLBCL (*n* = 414 training, *n* = 729 validation)	Consensus clustering, NB, DT, RF AB, XGB, SVM, Fisher discriminant analysis	Identified immune‐enriched and cell cycle‐enriched subtypes	External validation sets
He et al.^48^	DLBCL (*n* = 95 training, *n* = 72 validation)	Consensus clustering, SVM, Lasso, GBM, ridge regression, KNN, LR, LDA, DT, XGB, NB, elastic net regression, stepwise regression	Identified two subgroups based on programmed cell death‐related genes	External validation sets
Biccler et al.^49^	DLBCL (*n* = 2759 training, *n* = 2414 validation)	Stacked model	Developed a stacked model outperforming IPI and NCCN‐IPI in survival prediction	External validation set
Mosqueira Orgueira et al.^50^	DLBCL (*n* = 233 training, *n* = 64 validation)	mclust, RSF	Identified two clusters based on a 4‐gene signature with divergent survival rates	External validation set
Detrait et al.^51^	DLBCL (*n* = 130)	SVM, RF, LR, NB, XGB	F1‐score of 0.82 in predicting primary refractory patients	Internal cross‐validation
Zhao et al.^52^	DLBCL (*n* = 414 training, *n* = 855 validation)	DT, RF, SVM, NN, AB, Fisher discriminant analysis, Bagging	Identified 8 genes to predict COO with 91.0–94.4% accuracy	External validation sets
Viswanathan et al.^53^	DLBCL (*n* = 381)	MLP	Identified 20 genes to predict COO with 94.7%–95.9% accuracy	External validation set

Abbreviations: AB, adaptive boosting; DT, decision tree; GBM, gradient boosting machine; KNN, k nearest neighbour; LDA, linear discriminant analysis; LR, logistic regression; MLP, multi‐layer perceptron; NB, naïve Bayes; NN, nearest neighbour; RF, random forest; RSF, random survival forest; SVM, support vector machine; XGB, extreme gradient boosting.

### Follicular lymphoma and mantle cell lymphoma

Zha et al.^54^ developed an extreme gradient boosting (XGB) model (named FLIPI‐C) to predict POD24 using clinical and laboratory data of 1938 FL patients, achieving an AUC of 0.70 in an external validation set, reportedly outperforming both FLIPI and FLIPI2. The most predictive features in their model were lymphocyte/monocyte ratio, LDH, haemoglobin, β2‐microglobulin, PET SUV_max_ and nodal involvement. Using electronic health record (EHR) data from the Veterans Affairs Cancer Registry System, Li et al.^55^ developed a random survival forest (RSF) model integrating clinical and laboratory data among which albumin levels, age and erythrocyte count were the most important variables. Yet, their model was unable to outperform a Cox model based on POD24. Mosqueira Orgueira et al.^56^ used gene expression data of 184 FL patients to predict outcomes with an RSF‐based model named IAC‐FL. Best results were obtained when integrating their model with age and the m7‐FLIPI, achieving a c‐index of 0.79, reportedly outperforming m7‐FLIPI alone (c‐index 0.74). Using a neural net‐based approach, Carreras et al.^57^ identified 43 among 22 215 genes that were associated with favourable or unfavourable outcomes in FL. Highest ranking genes for favourable prognosis were *TDRD12* and *ZNF230* and *EVA1B, KRT19, BTN2A3P, KLK10, TRPM4, TMEM70* and *SLC24A2* for unfavourable prognosis. The same group conducted a similar study in mantle cell lymphoma (MCL),^58^ where their model identified *KIF18A, YBX3, PEMT, GCNA* and *POGLUT3* to be associated with poor outcomes while *SELENOP, AMOTL2, IGFBP7, KCTD12* and *ADGRG2* were associated with favourable outcomes. The authors report these alterations to be also prognostically relevant in external datasets of DLBCL and a multi‐entity cohort derived from The Cancer Genome Atlas. Using clinicopathological, genetic and laboratory data of 862 MCL patients, Hill et al.^59^ developed a gradient‐boosted model incorporating 20 features (named ‘integrative MIPI’ or iMIPI) with an ROCAUC of 0.82 and a simplified model (iMIPIs) with 10 features, achieving an ROCAUC of 0.81 in predicting relapse or progression after front‐line treatment with LDH, Ki67 and platelet counts being the top prognostic variables. Zhang et al.^60^ used recursive feature elimination to identify four immune‐related genes (*CD247, CD3E, CD4, GATA3*) delineating two clusters in 152 MCL patients with divergent outcomes. Table 6 summarizes studies investigating ML for risk prediction in FL and MCL.

**TABLE 6:** Studies investigating ML‐based outcome prediction in follicular lymphoma (FL) and mantle cell lymphoma (MCL).

References	Sample(s)	ML model(s)	Performance	Validation
Zha et al.^54^	FL (*n* = 1938 training, *n* = 1145 validation)	XGB, DT, RF, ridge classification	POD24 prediction, 0.70 ROCAUC in external validation	External validation set
Li et al.^55^	FL (*n* = 523)	RSF	Survival prediction, ROCAUC 0.73 (RSF did not outperform Cox model)	Internal cross‐validation
Mosqueira Orgueira et al.^56^	FL (*n* = 184 training, *n* = 138 validation)	RSF‐based model (named IAC‐FL)	Survival prediction, c‐index 0.79 when integrating IAC‐FL, m7‐FLIPI, and age	External validation set
Carreras et al.^57^	FL (*n* = 184)	MLP, radial basis function neural networks	Identified 25 favourable and 18 unfavourable prognostic genes	‐
Carreras et al.^58^	MCL (*n* = 123)	MLP, radial basis function neural networks	Identified 10 affecting prognosis also in DLBCL and a multi‐entity cohort	External validation sets of DLBCL (*n* = 414), multi‐entity (*n* = 7289)
Hill et al.^59^	MCL (*n* = 862)	XGB, RF	ROCAUC 0.81–0.82	Internal cross‐validation
Zhang et al.^60^	MCL (*n* = 152)	SVM, RF, Lasso, k‐means clustering	Identification of a 4‐gene signature defining two clusters with divergent OS	Internal cross‐validation

Abbreviations: DT, decision tree; MLP, multi‐layer perceptron; OS, overall survival; RF, random forest; ROCAUC, receiver‐operating‐characteristic area‐under‐the‐curve; RSF, random survival forest; SVM, support vector machine; XGB, extreme gradient boosting.

### Hodgkin lymphoma

A study by Rask Kragh Jørgensen et al.^61^ used a stacked model developed on data from the Danish National Lymphoma Register and validated on Norwegian and Swedish registers to predict PFS and OS in Hodgkin lymphoma (HL), achieving a c‐index of 0.665 and 0.749, respectively, thereby outperforming both IPS‐3 and IPS‐7 while being on par with A‐HIPI. DeAndrés‐Galiana et al.^62^ predicted treatment response (CR and PR) versus PD as well as CR versus PR in a cohort of 263 Spanish HL patients, obtaining accuracies of 95.8% and 92%, respectively, based on clinicopathological and laboratory data. Parodi et al.^63^ compared five ML models in predicting outcomes of 130 HL patients with the SVM model outperforming its competitors. Among selected predictive variables, they highlight an overexpression of *XIST* to be enriched in female patients and being associated with favourable outcomes. Patricio et al.^64^ used gene expression data from 106 HL patients to predict response after two cycles of ABVD, achieving an ROCAUC of 0.97 with an SVM classifier and identifying a set of 14 predictive genes. Similarly, Kuang et al.^65^ identified nine genes (*LAMP1, STAT1, MMP9, C1QB, ICAM1, CD274, CCL19, HCK* and *LILRB2*) associated with unfavourable prognosis using gene expression data from 130 HL patients, resulting in a predictive performance of ROCAUC 0.785 for 5‐year OS. Table 7 summarizes studies investigating ML for risk prediction in HL.

**TABLE 7:** Studies investigating ML‐based outcome prediction in Hodgkin's lymphoma (HL).

References	Sample(s)	ML model(s)	Performance	Validation
Rask Kragh Jørgensen et al.^61^	HL (*n* = 707 training, *n* = 760 validation)	Stacked model	Validation cohort: c‐index 0.749 (OS) and 0.665 (PFS)	External validation sets
deAndrés‐Galiana et al.^62^	HL (*n* = 263)	KNN	CR+PR vs. PD accuracy 95.8% CR vs. PR accuracy 92%	Internal cross‐validation
Parodi et al.^63^	HL (*n* = 130)	KNN, SVM, DT, neural net, Logic Learning Machine	Best‐performing model SVM, accuracy 81.5%	Internal cross‐validation
Patricio et al.^64^	HL (*n* = 106)	KNN, SVM, RF, XGB, DT, NB	Best‐performing model SVM, ROCAUC 0.97	Internal cross‐validation
Kuang et al.^65^	HL (*n* = 130)	Lasso, RF, LR	ROCAUC 0.785 (5‐year OS)	Internal cross‐validation

Abbreviations: DT, decision tree; KNN, k nearest neighbour; LR, logistic regression; NB, naïve Bayes; RF, random forest; ROCAUC, receiver‐operating‐characteristic area under the curve; SVM, support vector machine; XGB, extreme gradient boosting.

### Chronic lymphocytic leukaemia

In chronic lymphocytic leukaemia (CLL), multiple studies investigated ML models to predict time‐to‐first treatment (TTFT).^66^, ^67^, ^68^, ^69^ Chen et al.^66^ used clinical data, laboratory values and standard genetic markers to predict TTFT, reporting high performance for both RF and RSF, albeit that no model outperformed a standard Cox model. Meiseles et al.^67^ developed a model trained on a reduced set of clinical and laboratory data and found that the addition of genetic and flow cytometry data did not substantially boost TTFT predictive performance in their sample. Morabito et al.^68^ used autoencoders on gene expression data and identified *IGF1R* and *QTRT1* to be significantly associated with TTFT in multivariable analysis, resulting in a c‐index of 0.79 for their final model also including CLL‐IPI relevant variables. Similarly, Mosquera Orgueira et al.^69^ identified 19 genes that clustered CLL patients into two groups with different TTFT and developed a tree‐based classifier predicting the need for treatment within 5 years after diagnosis with 89% accuracy. Parviz et al.^70^ reported that the addition of clinical variables to CLL‐IPI improved outcome prediction for treatment initiation, occurrence of infection or death, while the addition of recurrent genetic lesions did not improve performance. In an ensemble learning approach, Agius et al.^71^ developed CLL‐treatment infection model (CLL‐TIM) on data from the Danish National CLL Registry, predicting treatment initiation or infection within 2 years with a precision and recall of 0.72 and 0.75 respectively. Coombes et al.^72^ used unsupervised learning to identify patient clusters with divergent survival and reported mutated immunoglobulin heavy‐chain variable region gene (*IGHV*) status, absent Zap 70 expression, female sex and younger age to be associated with favourable outcomes. Using flow cytometry data, Hoffmann et al.^73^ identified 17 distinctive cell populations in 157 CLL patients, of which a population enriched for CD4+ cells had the highest classification performance (ROCAUC 0.83) for outcome prediction. Table 8 summarizes studies investigating ML for risk prediction in CLL.

**TABLE 8:** Studies investigating ML‐based outcome prediction in chronic lymphocytic leukaemia (CLL).

References	Sample(s)	ML model(s)	Output	Validation
Chen et al.^66^	CLL (*n* = 737)	RF, RSF, LR, SVM, GBM	TTFT prediction c‐index RSF 0.93 (1 year) RF 0.92 (5 years)	Internal cross‐validation
Meiseles et al.^67^	CLL (*n* = 109)	GBM	TTFT prediction (2 years) AUPRC 0.78 (extensive) 0.77 (reduced)	Internal cross‐validation
Morabito et al.^68^	CLL (*n* = 217)	Autoencoder	Identified *IGF1R* and *QTRT1* as predictors of TTFT, c‐index 0.79	Internal cross‐validation
Mosquera Ogueira et al. ^69^	CLL (*n* = 196 training, *n* = 98 validation)	mclust, boosted trees	Identified 19 genes as predictors of TTFT, accuracy 89%	External validation
Parviz et al.^70^	CLL (*n* = 314)	AB, XGB, LightGBM, CatBoost, RF, ExtraTrees, SVM	Prediction of treatment initiation, infection, or death with CLL‐IPI, clinical variables; no added benefit of genetics	Internal cross‐validation
Agius et al.^71^	CLL (*n* = 3270)	Ensemble model named CLL‐treatment‐infection‐model (‐TIM)	Prediction of treatment initiation or infection, precision 0.72 recall 0.75	External validation
Coombes et al.^72^	CLL (*n* = 247)	k medoids	Clusters with divergent survival, favourable outcomes associated with mutated IGHV, absent ZAP70 expression, female sex, younger age	‐
Hoffmann et al.^73^	CLL (*n* = 157)	Algorithmic population descriptions (ALPODS)	Identified 17 populations in multi‐colour flow cytometry, ROCAUC 0.83 for a CD4+ enriched population	Internal cross‐validation

Abbreviations: AB, adaptive boosting; GBM, gradient boosting machine; LR, logistic regression; RF, random forest; RSF, random survival forest; SVM, support vector machine; XGB, extreme gradient boosting.

### Multiple myeloma

To predict undetectable measurable residual disease (MRD) in multiple myeloma (MM), Guerrero et al.^74^ used cytogenetics, tumour burden and immune biomarkers, achieving an accuracy of 0.72 in external validation. Using a transformer‐based model, Hussain et al.^75^ predicted PFS, OS and occurrence of adverse events (AE) outperforming the International Staging System (ISS) and with comparable performance to an RSF‐based model and DynamicDeepHit (a neural network) and predicted longitudinal biomarker trajectories, outperforming other tested neural network models. Using clinical, standard genetic and laboratory data from 338 elderly MM patients, Bao et al.^76^ compared neural networks—DeepSurv and DeepHit—to an RSF and standard Cox model, reporting comparable performances in predicting OS. Using standard prognostic laboratory values, Farswan et al.^77^ propose a model with alternative thresholds for albumin, calcium, glomerular filtration rate (GFR) and haemoglobin using k‐adaptive partitioning, reportedly outperforming RISS scores. Mosquera Orgueira et al.^78^ used clinical and gene expression data to develop a 50‐variable RF model, predicting OS with a c‐index of 0.78, incorporating age, ISS stage, β2‐microglobulin, first‐line treatment and expression of 46 genes, prominently involving cell cycle regulation and ubiquitin conjugation. In another study by the same group,^79^ unsupervised clustering was applied to clinical, laboratory and standard cytogenetic data of 730 MM patients, identifying two clusters with divergent outcomes restratifying patients from ISS and R‐ISS categories while only slightly outperforming both. Venezian Povoa et al.^80^ used gene expression data and cytogenetics to predict treatment sensitivity, reporting an AUC of 0.69 for their proposed multi‐learning training (MuLT) model. Borisov et al.^81^ used gene expression data of patients receiving bortezomib‐based regimens to identify *FGFR3*, *MAF*, *IGHA2*, *IGHV1‐69* and *GRB14* upregulation as markers of favourable response. Katsenou et al.^82^ used proteomic profiles of 39 MM patients and unsupervised clustering to screen for drug vulnerabilities, reporting CD9, ITB3 and F13A to be the top three most significant markers. To predict severe infections in daratumumab‐based regimens based on routine clinical and laboratory data, Mikulski et al.^83^ identified haemoglobin level and ECOG to be significantly associated with severe infections in multivariable analyses, reporting an ROCAUF of 0.87 for gradient boosting and RF respectively. Table 9 summarizes studies investigating ML for risk prediction in MM.

**TABLE 9:** Studies investigating ML‐based outcome prediction in multiple myeloma (MM).

References	Sample(s)	ML model(s)	Output	Validation
Guerrero et al.^74^	MM (*n* = 301 training, *n* = 186 validation)	LR	Prediction of undetectable MRD, accuracy 0.72	External validation
Hussain et al.^75^	MM (*n* = 703 training, *n* = 720 validation)	Transformer model (named SCOPE), DynamicDeepHit, RSF	Prediction of PFS, OS, adverse events, biomarker trajectories	External validation
Bao et al.^76^	MM (*n* = 338)	RSF, DeepSurv, DeepHit	OS prediction, c‐indexes 0.77–0.80	Internal cross‐validation
Farswan et al.^77^	MM (*n* = 716 training, *n* = 1254 validation)	k adaptive partitioning, BIRCH clustering, J48 classifier	Modified Risk Staging (MRS) model, introduces alternative thresholds for prognostic laboratory values	External validation
Mosquera Orgueira et al.^78^	MM (*n* = 730)	mclust, RF	Prediction of OS, c‐index 0.78	Internal validation
Mosquera Orgueira et al.^79^	MM (*n* = 752)	mclust	Identification of two risk clusters, c‐indexes 0.59–0.65 stratified by age	Internal cross‐validation
Venezian Povoa et al.	MM (*n* = 1525)	LightGBM, MLP, SVM, Multi Learning Training (MuLT)	Prediction of treatment sensitivity, AUC 0.69	Internal cross‐validation
Borisov et al.^81^	MM (*n* = 53)	SVM, RR, RF, NB, MLP	Identified *FGFR3*, *MAF*, *IGHA2*, *IGHV1‐69* and *GRB14* upregulation as prognostically favourable for bortezomib‐based regimens	Internal cross‐validation
Katsenou et al.^82^	MM (*n* = 39)	k means, LR, RF, SVM	Screening proteomic profiles for drug vulnerabilities	Internal validation
Mikulski et al.^83^	MM (*n* = 139)	Gradient boosting, RF, J48 classifier	Prediction of severe infections, ROCAUC 0.87	Internal cross‐validation

Abbreviations: BIRCH, balanced iterative reducing and clustering using hierarchies; GBM, gradient boosting machine; LR, logistic regression; MLP, multi‐layer perceptron; RF, random forest; RR, ridge regression; RSF, random survival forest; SVM, support vector machine; XGB, extreme gradient boosting.

## DISCUSSION AND OUTLOOK

ML models may soon enable data‐driven personalized outcome predictions using complex and high‐dimensional data in haematology. Yet, several challenges remain hindering widespread routine use. Any AI risk prediction model should be benchmarked against existing models in clinical routine as AI models not necessarily outperform conventional statistical models. Common limitations of current ML‐based outcome classifiers in haematology include their predominantly retrospective design and reliance on single institution training data with small sample size and high class imbalance. They often incorporate only one ML model, therefore lacking comparisons to other ML models or conventional statistical models, often include only a single data modality and lack external or prospective validation and standardization in reporting. Furthermore, training data and model parameters are seldom made publicly accessible, hindering independent verification and clinical translation. To mitigate bias and ensure robust generalizability, models should be trained on large, diverse datasets and validated on external cohorts. However, a lack of digitization, disincentives to data sharing and privacy concerns currently hinder the development of models on a large scale. These may be overcome using co‐operative learning strategies such as federated^84^ or swarm learning,^17^ centralized data collection efforts such as the Harmony Alliance^85^ or alternatively by sharing synthetic samples that mimic the behaviour of real patient cohorts without compromising privacy.^86^ Additionally, training sets should include real‐world data and provide adequate representation of minority groups and patients with rare subtypes to mitigate bias and enable generalization to a broader patient population in routine clinical settings rather than being tailored to clinical trial collectives many models are currently trained on.^87^, ^88^, ^89^

Models trained with the explicit intent of acting as a clinical decision support tool should be rigorously assessed and evaluated. Therefore, transparent quality control and standardization of reporting on such models in medical literature are essential. Guidance can be found in the transparent reporting of a multivariable prediction model for individual prognosis or diagnosis (TRIPOD), initially developed for multivariate statistical models^90^ and recently updated for machine learning^91^ and LLMs^92^; TRIPOD provides a comprehensive checklist streamlining transparent reporting including data, analytical methods, model specifications, limitations and intended use as well as stakeholder involvement and open science considerations. Technological risk mitigation should be guided by incorporating ‘Good Machine Learning Practices’ as recently proposed by the Food and Drug Administration (FDA).^93^

Apart from outcome prediction, AI models have been successfully implemented in haematological diagnostics including evaluation of peripheral blood^94^ and bone marrow smears,^95^ histopathology^96^ and radiological imaging,^97^, ^98^ identifying genotype–phenotype links,^99^, ^100^ automating flow cytometry,^101^ supporting karyotype analysis^102^ and interpretation of genetic variants.^103^ Hence, diagnostic AI models may complement each other and support decision‐making models in clinical routine in the near future. As AI permeates the diagnostic and therapeutic workflow in haematology, clinical integration requires a digital infrastructure and a workforce trained in AI applications. The first necessitates seamless integration in laboratory and clinical information systems to enhance and not disrupt existing workflows. AI models should aggregate information in an existing digital environment to provide comprehensive and explainable decision support in a user‐friendly interface without increasing workload by requiring haematologists to enter redundant information into external software. Haematologists should be trained in interpreting model outputs and be aware of model‐dependent limitations. This requires incorporating AI literacy in medical school and residency, ideally interwoven with concrete use cases as they arise during medical training. Furthermore, incorporating AI literacy into continuing medical education is essential, given that rapid technological advances demand ongoing updates in user competencies.

Given the increasing complexity of data in haematology, data‐driven ML models may be well equipped to address the complex biological interactions present in haematological malignancies. As these models mature, they have the potential to provide data‐driven disease classification systems and risk‐adapted treatment support. Bridging the gap between science and clinical routine will depend on sustained collaboration, robust validation and regulatory guidance.

## AUTHOR CONTRIBUTIONS

J‐NE conceptualized the study. J‐NE and WW performed the literature search and wrote the initial draft of the manuscript. J‐NE created visualizations. JMM and TH critically reviewed and edited the manuscript. All authors approved the final version of the manuscript.

## FUNDING INFORMATION

No funding was received for this study.

## CONFLICT OF INTEREST STATEMENT

J‐NE declares consulting services for AstraZeneca, Novartis, Janssen. Furthermore, he holds shares in Cancilico; has received an institutional research grant by Novartis; and has received honoraria by Amgen, AstraZeneca, Janssen, Novartis and Pfizer. JMM declares consulting services for Janssen, Roche, Gilead, Abbvie, Jazz, Pfizer, Astellas, Novartis, AstraZeneca, Clycostem. Furthermore, he holds shares in Cancilico and Synagen; has received institutional research grants by Janssen, Jazz and Novartis; and has received honoraria by Novartis, Roche, Janssen, Abbvie, Pfizer, Sanofi, Astellas and Beigene. TH is part owner of MLL Munich Leukemia Laboratory. WW is employed by MLL Munich Leukemia Laboratory.

## Data Availability

Data sharing not applicable to this article as no datasets were generated or analysed during the current study.
